# Ac-DEVD-CHO (caspase-3/DEVDase inhibitor) suppresses self-incompatibility–induced programmed cell death in the pollen tubes of petunia (*Petunia hybrida* E. Vilm.)

**DOI:** 10.1038/s41420-024-01821-x

**Published:** 2024-01-30

**Authors:** Ekaterina Vladimirovna Zakharova, Ilya Sergeevich Demyanchuk, Denis Sergeevich Sobolev, Yaroslav Yurievich Golivanov, Ekaterina Nikolaevna Baranova, Marat Rushanovich Khaliluev

**Affiliations:** 1 All-Russia Research Institute of Agricultural Biotechnology, 127550 Timiryazevskaya 42, Moscow, Russia; 2 Timiryazev Institute of Plant Physiology, Russian Academy of Sciences, 127276 Botanicheskaya 35, Moscow, Russia

**Keywords:** Proteases, Cell death, Plant cell biology, Reproductive biology

## Abstract

Programmed cell death (PCD) is relevant to many aspects in the growth and development of a plant organism. In their reproduction, many flowering plant species possess self-incompatibility (SI), that is an intraspecific reproductive barrier, which is a genetic mechanism ensuring the avoidance of inbreeding depression by preventing self-pollination. This phenomenon enhances intraspecific variation; however, SI is rather a hindrance for some fruit plant species (such as plum, cherry, and peer trees) rather than an advantage in farming. PCD is a factor of the S-RNase–based SI in *Petunia hybrida* E. Vilm. The growth of self-incompatible pollen tubes (PTs) is arrested with an increase in the activity of caspase-like proteases during the first hours after pollination so that all traits of PCD—plasma membrane integrity damage, DNA degradation/disintegration, and damage of PT structural organization (absence of vacuoles, turgor disturbance, and separation of cell plasma membrane from the cell wall)—are observable by the moment of PT growth arrest. We succeeded in discovering an additional cytological PCD marker, namely, the formation of ricinosomes in self-incompatible PTs at early stages of PCD. SI is removable by treating petunia stigmas with Acetyl-Asp-Glu-Val-Asp-aldehyde (Ac-DEVD-CHO), an inhibitor of caspase-3/DEVDase, 2 h before a self-incompatible pollination. In this process, the level of caspase-3-like protease activity was low, DNA degradation was absent, PTs grew to the ovary, fertilization was successful, and full-fledged seeds were formed.

## Introduction

Plants utilize programmed cell death (PCD) in many aspects of their life activities both in the norm and under stress conditions [1, 2]. Plant PCD has a set of specific cytological, physiological, and biochemical features distinguishing it from the PCD in animals. The orthologs of many key animal apoptotic genes, including the genes coding for caspases, are absent in the plant genome; however, the proteases with a caspase-like enzyme activity were shown to be necessary for the plant PCD (for review, see ref. [3, 4]). The role of caspase-3-like protease/DEVDase has been especially intensively studied; correspondingly, this enzyme activity is currently used as a PCD marker (for review, see ref. [5]).

So far, the experimental data have been accumulated suggesting that caspase inhibitors can suppress cell death in plants [5, 6]. In particular, there is the evidence that Acetyl-Asp-Glu-Val-Asp-aldehyde (Ac-DEVD-CHO), a caspase-3 inhibitor, blocks PCD in plants [7, 8]. Self-incompatibility (SI) is shown to trigger a cascade of responses that lead to PCD in the *Papaver rhoes* incompatible pollen, which includes the activation of a caspase-3-like protease [9]. Bosch and Franklin-Tong [8] reported a temporal and spatial SI-induced activation of caspase-like proteases (CLPs) in poppy pollen. The authors also report that SI activates VEIDase and LEVDase and that VEIDase is also involved in the SI-induced PCD. DEVDase and VEIDase are rather rapidly activated, being detectable as early as 1–2 h after the SI induction, while LEVDase activity reaches its maximum later. The SI-induced DEVDase can be localized to the cytosol and nucleus. Later, Lord [10] showed that an in vivo inhibition of YVADase activity prevented PCD and actin reorganization in the Madagascar lace plant.

The Solanaceae type SI emerged approximately 90 million years ago [11] and got its name because was for the first time discovered in species of this plant family [12]. This system was later observed in Scrophulariaceae and Rosaceae [13]. In addition, it was recently reported for representatives of the Rutaceae (pomelo) family [14]. The female determinant is S-RNases, extracellular proteins that accumulate in the style conductive tracts, mainly in its upper part, where the growth of self-incompatible PTs is arrested (for review, see ref. [15]). Depending on a particular family, the male determinant, F-box protein, is referred to as S-locus F-box protein (SLF) or S-locus F-box (SFB). Their genes are expressed in pollen. This protein was for the first time discovered in *Antirrhinum* [16] and later in *Prunus* [17], *Petunia* [18], and *Citrus* [14]. Thus, the mechanism underlying the Solanaceae type SI utilizes an S-RNase–mediated RNA degradation of self-incompatible PTs; in this process, S-RNase acts as a female determinant and SLF (or SFB) serves as a male determinant. However, manifold other external and internal factors may also influence pollen rejection/acceptance.

We have earlier shown that PTs die in pistil tissues via PCD during the operation of gametophytic SI in *Petunia hybrida* E. Vilm. [19]. Different methods, including TUNEL analysis, have detected the PCD markers, such as DNA fragmentation in the in vivo developing self-incompatible petunia PTs, and the activity of CLPs in SI-induced PCD. SI can be regarded as the trigger of the signaling cascade that leads the cells of a self-incompatible PT to PCD [20].

The growth of self-incompatible petunia PTs is arrested with an increase in the CLP activities during the first hours after pollination [21]. Using intravital visualization of caspase-3,7-like proteases, we have shown that CLPs are activated directly in self-incompatible PTs rather than in the adjacent pistil tissues [21, 22]. The CLP activities increase during the first 2–4 h after a self-incompatible pollination to further drop so that all signs of PCD are observable 8 h after pollination by the moment the PT growth is arrested [19].

In this study, we succeeded in identifying one more cytological marker of PCD, namely, the presence of ricinosomes at the early stages of SI-induced PCD. The main goal of this work was to suppress SI in petunia by inhibiting PCD in self-incompatible PTs. We succeeded in doing this by treating caspase-3-like protease/DEVDase with its inhibitor Ac-DEVD-CHO. In this process, the activity of caspase-3-like protease in self-incompatible pollination remained low, while PTs grew to the ovary, fertilization occurred, and full-fledged seeds were formed.

## Results

### Morphology and ultrastructure of PT cell in pistil tissues

**Fig. 1:**
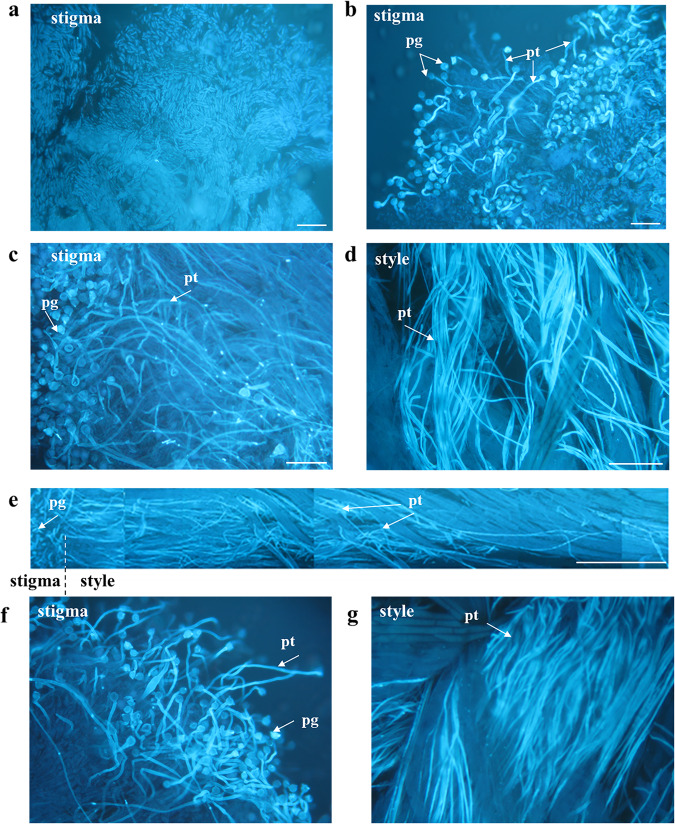
Aniline blue staining of petunia pollen tubes growing in vivo in pistil tissues. **a** Unpollinated stigma of a pistil. **b**–**e** Control – self-incompatible pollination (without Ac-DEVD-CHO caspase-3 inhibitor treatment). **f**, **g** Experimental - self-incompatible pollination after Ac-DEVD-CHO caspase-3 inhibitor treatment. **b** Pollen grains and pollen tubes on stigma surface (2 h after pollination); **c**, **f** Pollen grains and pollen tubes on stigma surface (4 h after pollination). **d**, **g** Pollen tubes growing in style tissues (8 h after pollination); bar = 100 μm. **e** Panorama of growing pollen tubes in pistil tissues; bar = 1000 μm. Abbreviations: pg, pollen grain; pt, pollen tube.

**Fig. 2:**
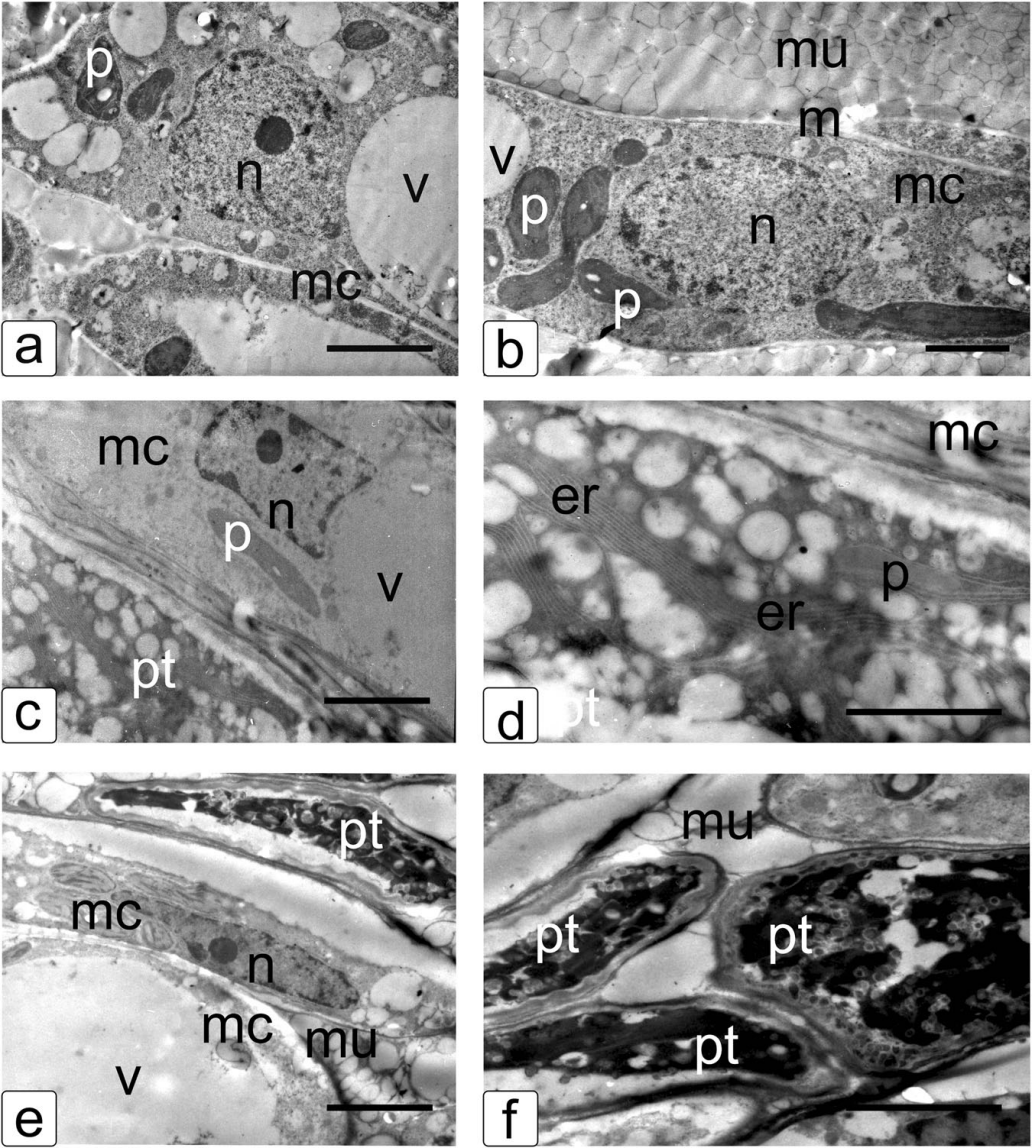
Transmission electron microphotographs of petunia style transmitting tract tissues with pollen tubes growing in them. **a**, **b** The cells of style transmitting tract tissue before pollination. Note compact cells, the cytoplasm enriched with organelles, numerous starch grains in chloroplasts, and a cell with a large vacuole. **c**, **d** The cells of style transmitting tract tissue and pollen tubes after a cross-compatible pollination. Note well-developed vacuoles and other cell components. **e**, **f** Style tissue and pollen tube cells after a self-incompatible pollination. Note degraded inner structure of pollen tube, the absence of vacuoles, turgor disturbance, and separation of cell plasma membrane from the cell wall. Abbreviations: n, nucleus; pt, pollen tube; v, vacuole; mc, mother cells (style transmitting tract tissues); m, mitochondria; p, plastid; mu, mucus; and er, endoplasmic reticulum. The SI response in petunia pollen tubes causes destruction of their inner structure. Bar = 1 μm.

Visualization of petunia pollen tubes growing in transmitting pistil tissues with aniline blue staining (Fig. 1) and transmission electron microscopy (Fig. 2) showed that after both cross-compatible and self-incompatible pollinations, almost all pollen grains appeared to be germinated and pollen tubes grew in stigma and style tissues (Fig. 1d, e). Figure 1 shows the growth of self-incompatible pollen tubes in petunia pistil tissues at different hours of pollination. It is visually impossible to distinguish the difference when stained with aniline blue between the control option (without Ac-DEVD-CHO caspase-3 inhibitor treatment) (Fig.1c, d) and the experimental option (after Ac-DEVD-CHO caspase-3 inhibitor treatment) (Fig.1f, g). In the case of self-incompatible pollination, pollen tubes stop growing approximately 8 mm from the surface of the stigma (Fig. 1e); after treatment, pollen tubes grow to the ovary, where fertilization occurs.

However, the PTs after a self-incompatible pollination also develop through the conductive tissues of pistil but cease growing 6–8 h after pollination at a distance of 8–9 mm from the stigma surface (Fig. 1e).

Figure 2a shows the cells of the transmitting tract of petunia unpollinated pistil. Cells are large, with nuclei in the center of the cell and well-structured cytoplasm with numerous ribosomes, plastids, and mitochondria. The vacuoles are uniformly distributed in the cytoplasm; plastids have a complex lamellar structure and starch inclusions. A large amount of mucus is distributed in between cells; the mucus is represented by numerous bubbles filled with the substances providing the growth of PTs.

Figure 2b, c shows an active growth of PTs 12 h after cross-compatible pollination. The cytoplasm in PT cells is denser as compared with the cells of pistil conductive tract and carries numerous inclusions and vacuoles as well as pinocytic structures, providing their growth. Flattened cisterns of the endoplasmic reticulum (ER) forming a network that run through the entire cytoplasm of PT cells are also observable (Fig. 2d).

Dead PT cells with lost turgor are observable between mother cells 12 h after a self-incompatible pollination (Fig. 2e, f). The cytoplasm has an electron-dense structure is considerably compacted and partially fragmented and its ultrastructure is disturbed. This is accompanied by compaction of the remaining cytoplasm in which fine structures are indistinguishable. The signs of irreversible lesions characteristic of PCD are present at this stage. Note that the pistil mother cells retain their native structure and abundant surrounding mucus (Fig. 2e).

**Fig. 3:**
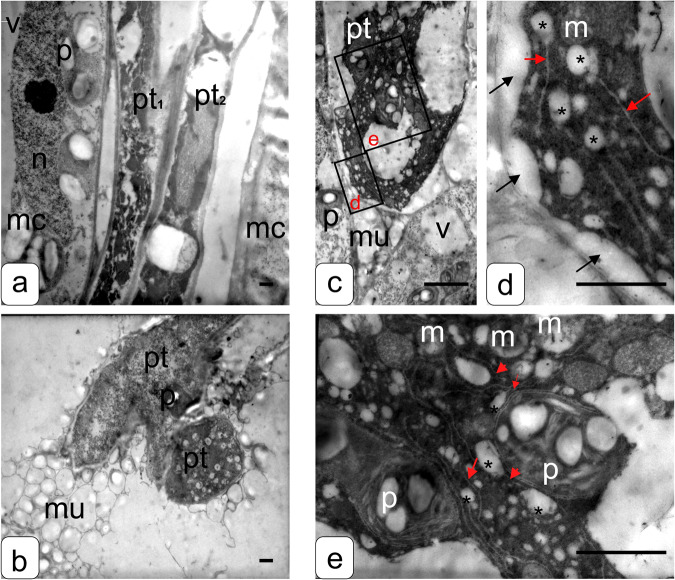
Transmission electron microphotographs of petunia style with transmitting tract tissues and pollen tubes growing in them (4 h after pollination). **a**, **b** Style tissue and pollen tube cells after a self-compatible pollination. Pollen tube cell contains well-developed vacuoles and other cell components. **c–e** Style transmitting tract tissue and pollen tube cells after a self-incompatible pollination. The cytoplasm has an electron-dense structure. It is noticeably compacted; tonoplast is separated from the cell wall. This pattern probably reflects differences in osmotic pressure and usually accompanies a difference in turgor; a large amount of modified ER with forming along the edges dilated fragments, having a spherical shape corresponding to ricinosomes is formed. Abbreviations: pt, pollen tube; mc, mother cells (style with transmitting tract tissues); m, mitochondria; p, plastid; red arrow, endoplasmic reticulum; asterisk, ricinosome-like body; black arrows, separation of the cytoplasmic membrane; mu, mucus; and v, vacuole. Bar = 1 μm.

Quite different picture of developing PTs is observable in the pistil conductive tissues 4 h after a self-incompatible pollination (Fig. 3). Fluorescence microscopy shows that the length of self-incompatible PTs by this moment reaches 4125 ± 126 μm (Fig. 1c). As is evident from Fig. 3, morphological changes in self-incompatible PTs accompany their development and are reflected in the ultrastructure of cells in the early stages of PTs growth.

Figure 3a, b shows developing PTs. Note considerably structured cytoplasm with individual foci of endocytosis, providing intensive PT growth (Fig. 3B), in pt_1_. The ongoing fragmentation of the cytoplasm is observable in pt_2_ as well as the formation of compartments—vacuoles and callose plugs that separate the growing PT tip from the nonfunctional upper part.

PT cells with well organized cytoplasm and numerous compartments—vesicles characteristic of growing PTs and surrounded by mucus are evident in cross-section (Fig. 3b). The cell membrane in this part PT tightly fits the tonoplast, demonstrating an increased pressure that provides its growth.

In the case of 4-h self-incompatible pollination (Fig. 3c–e), considerable compaction of the cytoplasm (appearing as a homogeneous unstructured osmiophilic zone), separation of the tonoplast from the cell membrane, and formation of a large amount of modified ER with dilated spherical extensions at the end corresponding to ricinosomes, observable in a number of cases of plant PCD, are seen. The ricinosome-like structures reside in dense cytoplasm and are spherical, formed of membranes, and uniformly spread in between cell organelles. Plastids preserve individual elements of ultrastructure, for example, starch grains and lamellar structures. The stroma of plastids is dense. On the contrary, mitochondria have a bright matrix and increased sizes (swollen). The cristae are retained but nonuniformly distributed; individual ribosomes are detectable as well as filamentous structures (presumably, DNA). It is evident that cells retain viability and compartmentalization. Thus, changes in osmosis, disturbances in the density of individual organelles, and the appearance of ricinosome-like bodies indicate that the cells of self-incompatible PTs display the signs matching the progress in PCD (Fig. 3c–e).

### Ac-DEVD-CHO suppresses the SI-induced PCD in self-incompatible petunia pollen tubes

#### Effects of Ac-DEVD-CHO on in vivo pollen tube growth

The visualization (staining with aniline blue) of the PTs developing in petunia pistil tissues demonstrates that almost all pollen grains after cross-compatible and self-incompatible pollinations successfully germinate and the PTs grow in the stigma and upper style part (Fig. 1). In the case of a compatible pollination, the PT length 24 h after plant was 17 322 ± 266 μm, which is 80.96% of the pistil length. In the case of a self-incompatible pollination, the growth of PTs is arrested 6–8 h after pollination at a distance of approximately 8–9 μm from the stigma surface and remains at this level 24 h after plant as well (Tables 1 and 2).

**Table 1:** The effect of Ac-DEVD-CHO (0.25 mM) treatment of the stigmas of self-incompatible petunia line on the growth of self-incompatible PTs (μm): self-incompatible pollination.

Time after pollination	Self-incompatible pollination			
	Control	Stigmas are treated with Ac-DEVD-CHO		
		2 h before pollination	Simultaneously with pollination	2 h after pollination
4 h	3 501 ± 123	415 ± 23*	3 123 ± 215	1 133 ± 111*
6 h	4 125 ± 126	3 238 ± 167*	5 516 ± 165*	4 222 ± 187
24 h	9 123 ± 344	15 123 ± 123*	13 553 ± 233*	14 311 ± 314*

^*^Treatments that are significantly different from the control at *P* < 0.05 are marked with an asterisk.

**Table 2:** The effect of Ac-DEVD-CHO (0.25 mM) treatment of the stigmas of self-incompatible petunia line on the growth of compatible PTs (μm): cross-compatible pollination.

Time after pollination	Compatible pollination			
	Control	Stigmas are treated with Ac-DEVD-CHO		
		2 h before pollination	Simultaneously with pollination	2 h after pollination
4 h	4 330 ± 154	2 532 ± 88*	2 506 ± 164*	3 233 ± 203*
6 h	4 758 ± 381	3 238 ± 167*	6 123 ± 213*	4 325 ± 216
24 h	17 322 ± 266	17 522 ± 333	17 345 ± 344	12 516 ± 123*

^*^Treatments that are significantly different from the control at P < 0.05 are marked with an asterisk.

In this study, the PT length in the control variant 24 h after a self-incompatible pollination was 9 123 ± 344 μm, amounting to 31.9% of the length of pistil (Table 1).

For treating petunia stigmas, we used Ac-DEVD-CHO, a caspase-3 inhibitor, at four concentrations.

1. Treatment with Ac-DEVD-CHO at a concentration of 1.99 mM was fatal for petunia pistils in every variant of pollination (2 h before pollination, simultaneously with pollination, and 2 h after pollination). Pistils darkened, the stigmas withered, and pollen failed to germinate;
2. The concentration of 1 mM considerably inhibited the growth of both compatible and self-incompatible PTs (in every variant of pollination); their length did not exceed 7–9% of the pistil length by 24 h after pollination;
3. The concentration of 0.5 mM was not so dramatic for the PT development. The treatment with the caspase-3 inhibitor 2 h after pollination slowed down the growth of compatible PTs to 6.85% of the pistil length and of self-incompatible PTs, to 2.07%. The treatment 2 h before pollination had no effect on the growth of compatible PTs: their length was comparable to the control (17 565 ± 413 μm), while the growth of self-incompatible PTs was inhibited to 2 333 ± 134 μm. The treatment simultaneous with pollination inhibited the growth of compatible PTs to 14 344 ± 403 μm and somewhat stimulated self-incompatible PTs (to 10 105 ± 630 μm); and
4. The Ac-DEVD-CHO concentration of 0.25 μm gave the most significant results: the development of self-incompatible PTs increased almost to the level of compatible PTs (Table 1) in all variants of the treatment 2 h before pollination, simultaneously with pollination, and 2 h after pollination. Moreover, this concentration had almost no effect on the growth of compatible PTs (Table 2).

Pollinated pistils were left for assessing the rate of seed setting in all variants of the treatment. The treatment with the inhibitor at concentrations of 0.5 and 0.25 mM did not interfere with the normal rate of seed setting in all variants of compatible pollinations.

**Fig. 4:**
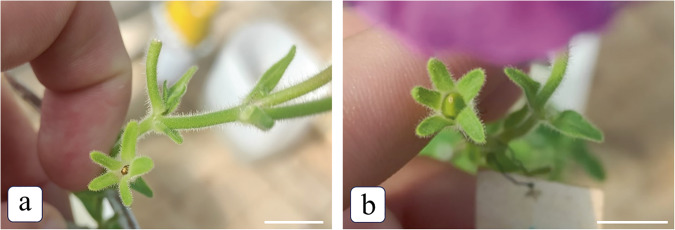
Effects of Ac-DEVD-CHO on the in vivo growth of self-incompatible pollen tubes. **a** Self-incompatible pollination (control): ovary is not formed and seeds do not set. **b** Self-incompatible pollination after the treatment with AC-DEVD-CHO, a caspase-3 inhibitor, at a concentration of 0.25 mM 2 h before pollination: a seed case set after a self-incompatible pollination after the treatment. Bar = 1 cm.

Note that only one variant of the treatment with Ac-DEVD-CHO, namely, a concentration of 0.25 mM 2 h before pollination, led to a 100% rate of seed setting in a self-incompatible pollination (Fig. 4).

#### DNA degradation

DNA degradation and caspase-3-like protease activities in petunia pistils were the markers of PCD in this study.

**Fig. 5:**
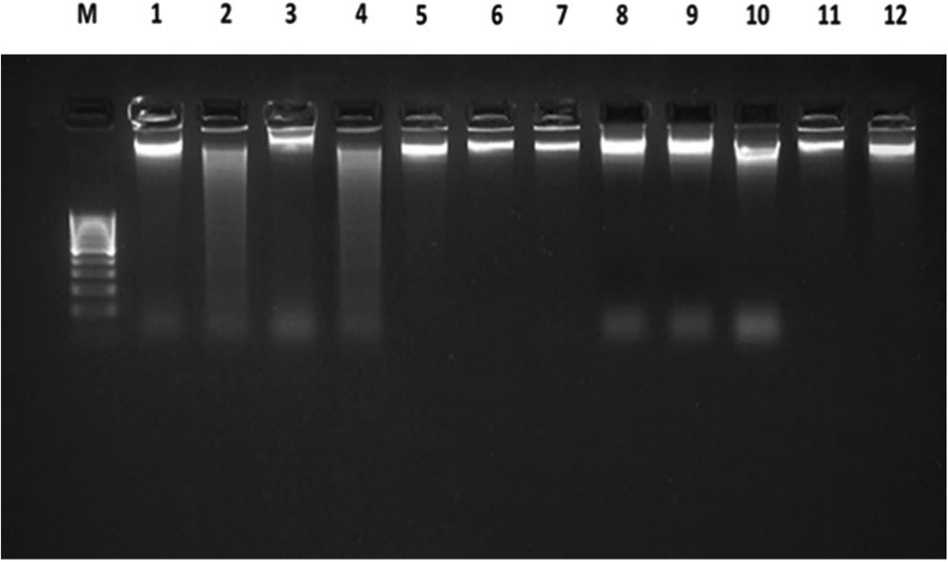
Electrophoresis of DNA isolated from the pistils (stigma and style) of self-incompatible petunia line treated with caspase inhibitor (Ac-DEVD-CHO). **М**, 1 kb DNA Ladder (Evrogen, Russia); **1**, unpollinated pistils from buds with anthocyanin of self-incompatible line petunia ( − DNA degradation), negative control; **2**, anthers from buds without anthocyanin of self-incompatible line petunia ( + DNA degradation), positive control; **3**, pistils 6 h after cross-compatible pollination ( − DNA degradation); **4**, pistils 6 h after self-incompatible pollination ( + DNA degradation); **5**, pistils 6 h after cross-compatible pollination + 0.25 mM inhibitor (Ac-DEVD-CHO) 2 h before pollination; 6, pistils 6 h after cross-compatible pollination + 0.25 mM inhibitor (Ac-DEVD-CHO) at the moment of pollination; **7**, pistils 6 h after cross-compatible pollination + 0.25 mM inhibitor (Ac-DEVD-CHO) 2 h after pollination; **8**, pistils 6 h after self-incompatible pollination + 0.25 mM inhibitor (Ac-DEVD-CHO) 2 h before pollination; **9**, pistils 6 h after self-incompatible pollination + 0.25 mM inhibitor (Ac-DEVD-CHO) at the moment of pollination; and **10**, pistils 6 h after self-incompatible pollination + 0.25 mM inhibitor (Ac-DEVD-CHO) 2 h after pollination.

The use of electrophoretic analysis as a method for separation of DNA fragments has allowed us to detect the presence of DNA degradation in a sample 6 h after a self-incompatible pollination. Note that DNA degradation was absent in the case of a self-compatible pollination (Fig. 5). The samples of anthers from the buds without anthocyanin of the self-incompatible clone were used as a positive control of DNA degradation since it is known that the tapetum cells die via PCD during another development. However, DNA degradation was absent in both self-compatible and self-incompatible pollinations in the samples treated with the caspase-3 inhibitor at concentrations of 0.5 and 0.25 mM.

#### CLP activity in petunia pollen–pistil system during self-incompatible pollinations

The formation of a self-incompatible PT and its growth in the stigma and style for 4 h were accompanied by a sharp increase in the caspase-3-like protease activity. During this period, the caspase-3-like protease activity in the pollen–pistil system increased almost eightfold as compared with the growing compatible PTs [21]. A drastic decrease in the caspase-3-like protease activity was observed in the pollen–pistil system 4 h after pollination (Table 3). A sharp decrease in the level of caspase-3-like protease activity is observed in the pistils treated with Ac-DEVD-CHO caspase-3 inhibitor at a concentration of 0.25 mM 2 h before a self-incompatible pollination during the overall period after pollination (Table 3).

**Table 3:** Spectrophotometric estimation of the CLP activity in the petunia pollen–pistil system after self-incompatible (SI) pollinations and after the treatment with Ac-DEVD-CHO at a concentration of 0.25 mM 2 h before pollination (nmol/AMC/min/mg).

Time after pollination	Unpollinated pistil	2 h	4 h	6 h	24 h
Variant					
SI pollination (control - no treatment Ac-DEVD-CHO) + DEVDase substrate (Ac-DEVD-AMC)	123,8 ± 11,4	554,5 ± 39,1	595,2 ± 47,5	89,61 ± 9,1	0,51 ± 0,04
SI pollination after treatment with 0.25 mM Ac-DEVD-CHO 2 h before pollination + DEVDase substrate (Ac-DEVD-AMC)	123,8 ± 11,4	15,00 ± 9,4	28,86 ± 6,2	17,49 ± 6,7	24,89 ± 12,3

^*^Total protein was extracted from 10 pistils without ovary (~100 mg) and incubated with CLP/DEVDase substrate (Ac-DEVD-AMC). The data are shown as mean peptide cleavage activity rate (nmol AMC/min/mg); error bars show the standard error (*n* = 2).

#### Visualization of the SI-induced caspase-3/7-like protease activity in the in vivo growing pollen tubes

**Fig. 6:**
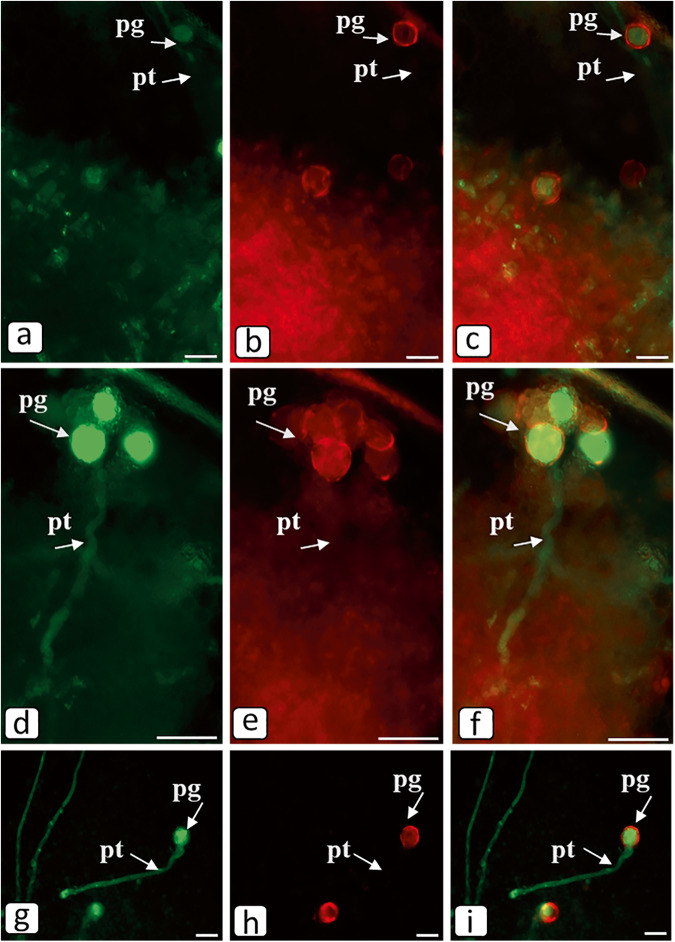
Caspase-3/7-DEVDase activity in the growing in vivo self-incompatible pollen tubes (2 h after a self-incompatible pollination). **a**–**c** After Ac-DEVD-CHO caspase-3 inhibitor treatment at a concentration of 0.25 mM 2 h before a self-incompatible pollination. **d**–**i** Without Ac-DEVD-CHO caspase-3 inhibitor treatment. **a**, **d**, **g** 488/530 nm FAM-DEVD-FMK caspase-3 and -7 reagent. **b**, **e**, **h** 535/617 nm propidium iodide. **c**, **f**, **i** FAM-DEVD-FMK caspase-3 and -7 reagent + propidium iodide (pg, pollen grain; pt., pollen tube; scale bar = 50 μm). The green fluorescence signal in the pollen grains and tubes indicated the presence of caspase-3 and caspase-7 activities in the cells.

Intravital imaging allowed us to experimentally demonstrate that the observed caspase-3/7-DEVDase activity in the petunia pollen–pistil system (Fig. 6) resides exactly in the self-incompatible pollen tubes rather than pistil cells. Green fluorescent signal in the pollen tubes indicates the presence of active caspases at the time of their detection. The activity of CLP was detected in 90% of the in vivo growing self-incompatible pollen tubes (2 h after a self-incompatible pollination; Fig. 6d–i). In the control self-incompatible pollination, we observe a characteristic bright green fluorescence signal. This is further demonstration that PCD enzymes are active within 2 h of self-incompatible pollination. The CLP activity was absent in the experimental variant (after Ac-DEVD-CHO caspase-3 inhibitor treatment at a concentration of 0.25 mM 2 h before a self-incompatible pollination) (Fig. 6a–c). Propidium iodide, is unable to enter the nuclei of a pollen tube, since it is not membrane-permeable and is used to differentiate apoptotic and healthy cells. Propidium iodide stains only the cell walls of living cells and the nuclei of dead cells with damaged plasma membrane. The dye stained red the outer cell wall of pollen grains, allowing for an accurate localization of the pollen tube in the pistil tissue, especially in the case of pollen tubes after Ac-DEVD-CHO caspase-3 inhibitor treatment (Fig. 6a–c). Thus, the self-incompatible pollen tubes at this stage (2 h after a self-incompatible pollination) are living and their plasma membrane retains its integrity, although the PCD process has already been triggered. The results suggest that the SI-induced CLP activity is localized to the cytoplasm in the self-incompatible pollen tubes. While treatment with Ac-DEVD-CHO caspase-3 inhibitor removes the CLP activity at this point. What once again proves the inhibition by Ac-DEVD-CHO caspase-3 inhibitor treatment of self-incompatibility–induced PCD in the pollen tubes of petunia.

Thus, our data demonstrate that the treatment with the caspase-3 inhibitor (Ac-DEVD-CHO) at a concentration of 0.25 mM 2 h before a self-incompatible pollination inhibits caspase-3 activity, prevents the DNA degradation characteristic of self-incompatible pollination at the moment when PT growth is arrested, increases the PT development to the level of compatible PTs thereby removing the phenomenon of SI, and is confirmed by setting of full-fledged seeds.

## Discussion

### S-RNase–based SI in *P. hybrida*

Many plant species possess the system of self-incompatibility, which prevents self-fertilization, enhances variation, and limits inbreeding [23]. However, SI as a hindrance to fertilization considerably interferes with an increase in yields, for example, of *Fragaria* [24, 25], *Citrus* [14, 26], and *Prunus* [27].

The incompatibility of sex elements during pollination in plants appears as the inability of PTs to grow through the entire pistil length and take part in fertilization although both pollen and pistil are completely functionally active. The SI in petunia (Solanaceae family) is regulated by polymorphic S locus, which comprises the S-RNase specific of the pistil and multiple F-box locus (SLF) genes specific of pollen [28]. SLF genes are assembled into the complexes known as E3 ubiquitin ligase complexes Skp1–Cullin1–F-box (SCFSLF). In PTs, these complexes jointly mediate ubiquitination and degradation of all foreign S-RNases but not the own S-RNase, which leads to a cross-compatible rather than a self-incompatible pollination [29].

In a self-compatible or a cross-compatible pollination, the petunia PTs grow through the conductive tissues of the pistil (Table 2) to reach the ovary, where fertilization takes place and seeds are set. In a self-incompatible pollination, (Table 1, Figs. 1, 2), the PT growth is arrested in the conductive tissues via the S-RNase–based SI and die via PCD [19, 21].

### PCD is a factor of S-RNase–based SI in *P. hybrida*

The SI-induced PCD of self-incompatible petunia PTs belong to dPCD, developmental PCD (for review, see ref. [30]). Several main PCD types are currently distinguished in plants, in particular, apoptosis-like PCD, autophagy [31], aging, and vacuole-mediated cell death or autolysis [2, 32]. As for the case of the death of self-incompatible petunia PTs, we may just assume that it is similar to an apoptosis-like PCD according to the morphological traits, such as plasma membrane integrity damage, DNA degradation/fragmentation, and damage of PT structural organization (absence of vacuoles, turgor disturbance, and separation of the cell plasma membrane from the cell wall) [19].

In this study, we have discovered ricinosome-like structures (Fig. 3). Ricinosome, precursor protease vesicle, is a relatively new organelle so far discovered only in plants [33]. Ricinosomes were for the first time found in 1970 by two independent teams and were described as “dilated cisternae” because they develop from the ER [34] and were referred to as ricinosomes being considered unique for the castor bean (*Ricinus communis*) [35]. Ricinosomes develop as ER dilations and can remain connected with it. This was described in several plant objects, in particular, maize [36] and mutant barley cultivar Nevskii [37]. Electron microscopy has shown that the structures of this type developing from the ER are observable in aging tissues. Biochemical examination demonstrate that they contain a large amount of the precursor of the 45 kDa cysteine protease with C-terminal KDEL motif.

The plant cells aging via PCD express the cysteine endopeptidases of a papain type with C-terminal KDEL sequence [38] as inactive enzymes with a molecular weight of 45 kDa, and are retained to the final PCD stage, when they are processed to the mature 35 kDa species with a 50–100-fold higher activity [39]. Acidification of isolated ricinosomes activates endopeptidases. It is assumed that ricinosomes accumulate during PCD-based aging and are activated by the release of protons from acidic vacuoles.

The cysteine endopeptidase of aging castor bean endosperm, Cys-EP, is homologous to the papain-type KDEL cysteine endopeptidases of the lower petals of aging daylily flowers, maturing French bean pods, and cotyledons of germinating black gram seeds and vetch [39, 40] and are stored in specialized organelles, ricinosomes [38, 39]. During the final collapse, ricinosomes are disorganized to release the mature enzyme. Acidification of the cytoplasm disintegrates ricinosomes in vivo, which changes the tonoplast permeability at the late PCD stage [4].

While studying the role of cytokinins during SI-induced PCD of self-incompatible PTs [21, 22], we hypothesized that a sharp increase in the concentration of cytokinins by the moment of growth arrest of self-incompatible PTs shifts the pH in the cytoplasm towards acid values, thereby creating the optimal conditions for CLPs. We have earlier demonstrated that cytokinins are the only phytohormones that drop the pH of PT cytoplasm to 5.5–5.8 [41]. Our latest data suggest that the shift in pH towards acid values is also necessary to disintegrate the membranes of ricinosomes and to release the mature enzyme at final PCD stages.

### CLP activity in the SI-induced PCD *in P. hybrida*

Intensive research into PCD over the last 30 years has clarified a large diversity in the types of PCD development even within the same organism. Many of them are morphologically described; however, the data on the underlying biochemical mechanisms are rather incomplete. The roles of some physiological factors, such as reactive oxygen species [42] and phytohormones [22, 43] have been revealed. However, there are no doubts that caspases are ideal effectors for providing PCD [44]. First, caspases are constitutively expressed as an inactive proenzyme. Thus, a certain amount of caspases is constantly present in the cell and can fulfill their function in sufficiently short terms [45]. Second, the proteolytic cleavage reactions catalyzed by caspases are irreversible [46]. Third, a considerable advantage of caspases consists in their multiple specificity, which, on the one hand, provides the cleavage of many substrates and, on the other hand, noncleavage of absolutely all proteins [47].

Two major groups of caspases are distinguished: (1) caspase-1, -4, -5, -13, and -14 and (2) the caspases that play the central role in apoptosis (caspase-2, -3, and -6–10). The latter group is subdivided into initiator (caspase-2, -8, -9, and -10) and effector (caspase-3, -6, and -7) enzymes [48]. Different mechanisms of apoptosis induction via membrane receptors or mitochondria meet at the level of caspase-3; correspondingly, the measurement of caspase-3 activity is among the most informative methods for the detection of apoptosis [49, 50]; finally, this particular method is responsible for most effects providing cell death [45].

Although the genes orthologous to animal caspases are absent in plants, at least eight caspase-like activities have been so far described; these activities are measured in plant extracts using caspase substrates [5, 6]. The importance of caspase-3 enzyme activity for the plant PCD has been comprehensively documented, in particular, with the help of caspase-3 inhibitors [5]. Correspondingly, we mainly work with the caspase-3-like protease DEVDase.

Many researchers report that the CLP activities are higher before visible PCD commencement. In particular, an early CLP activity is observable during cell autophagy in the common spruce and PCD in embryonic cell death, where in vivo studies demonstrate a DEVDase activity at the beginning of cell death stage [51]. Bosch et al. [52] discovered a CLP activity in self-incompatible *Papaver rhoeas* PTs during SI-induced PCD. We have also observed a burst of CLP activity during the first 2–4 h after a self-incompatible pollination (Table 3, Fig. 6), while a self-incompatible PT (DNA degradation/fragmentation) dies by 8 h after the pollination (Fig. 5). We also observe (Fig. 3) that any visible changes in the structure of PT cells are absent by 4 h after pollination except for the presence of detectable ricinosome-like structures. In this process, PTs grow, that is, all processes necessary for cell life activity and growth still go on there. In this (Fig. 6) and our earlier studies, we used intravital caspase-3,7-like protease visualization and demonstrated that their activities were determined directly in self-incompatible PTs rather than in the adjacent pistil conductive tissues [21, 22].

### Suppression of SI-induced PCD

In terms of evolution, a cross-pollination, which leads to the exchange of hereditary properties between cultivars at a genetic level, is a progressive phenomenon. However, it is often necessary to overcome SI in many species for farming, research, and breeding purposes. Several methods are available here, namely, pollination in buds, in vitro application of pollen onto the placenta, X-ray or ultraviolet irradiation of pollen, application of chemical mutagens and various substances, polyploidization, and so on.

As is mentioned above, any true caspases have not been discovered in plants; only their analogs have been identified [5]; and synthetic caspase inhibitors have been used to arrest PCD in both animal [53] and plant [5] objects. An activity similar to DEVDase/caspase-3 was identified in a number of plant systems using Ac-DEVD tetrapeptide inhibitors that suppress PCD [54, 55]; in addition, PCD was suppressed by cleaving Ac-DEVD-AMC, a caspase-3 specific substrate [56].

Specific inhibitors of animal caspases influence the development of PCD in plants [57]. In particular, animal caspase-1 and caspase-3 inhibitors YVAD-cmk and Ac-DEVD-CHO weakened the hypersensitive response induced by bacteria and tobacco mosaic virus in tobacco leaves [54]. The same sets of caspase inhibitors decreased the isopentyladenosine-induced death of tobacco cells [58] and were effective in the menadione-induced apoptosis in tobacco protoplasts [59]. The nitric oxide– and H_2_O_2_-induced PCD in arabidopsis suspension culture was suppressed by a caspase-1 inhibitor [60]. The PCD induced in the suspension cell culture of tobacco with the help of ethylene-inducing fungal xylanase elicitor was blocked with ZVAD-fmk and BocD-fmk, wide range caspase inhibitors [61]. Caspase or cysteine protease inhibitors injected into the remaining epicotyl tissue suppressed PCD of the secondary shoots giving the seedlings with two equal shoots [7]. Addition of caspase-3 (Ac-DEVD-CHO) or caspase-1 inhibitor (Ac-YVAD-CMK) suppressed a hypersensitive response of the cells to the PCD induced by avirulent bacteria or mycotoxins [62]. Caspase-specific inhibitors also effectively blocked the PCD induced by chemical substances and heat shock [59, 63].

In this study, we succeeded in removing the SI in petunia by the treatment with a CLP inhibitor (Ac-DEVD-CHO), which is confirmed by setting of full-fledged seeds (Fig. 4).

## Conclusions

In this study, we have shown that the protease(s) with a caspase-like activity play a decisive role in the SI-induced PCD in petunia (*P. hybrida* E. Vilm.) and discovered an additional signaling pathway mediating a SI response in this species. By inhibiting PCD by the treatment with a caspase-3-like protease (Ac-DEVD-CHO), we successfully suppressed the overall SI phenomenon, which is confirmed by setting of full-fledged seeds.

Further, this approach can serve as a new method for overcoming of SI to increase the propagation efficiency of inbred lines in agricultural practice. This can form the basis for the technology of seed and fruit production of the agricultural plants with a genetically determined SI barrier. Fruit trees are vegetatively propagated because SI requires that fruit gardens are multicultivar since a monocultivar variant makes such gardens fruitless (for example, cherries and plums). Self-pollinated (self-fertile) cultivars are better as compared with cross-pollinated and are able to set fruits in the case of adverse weather conditions and a decrease in insect pollinators caused by deterioration of ecological situation.

## Materials and methods

### Experimental design

#### Plant material

Clonally propagated *P. hybrida* E. Vilm. plants of self-compatible and self-incompatible lines from the laboratory collection were grown and adapted to soil conditions under natural illumination in a greenhouse. The clonal micropropagation was performed in the Murashige–Skoog medium; the microclones were cultivated in a climate chamber at a temperature of 26 °C and 16 h light/day.

The experiments were conducted in an in vivo pollen–pistil system after self-incompatible and cross-compatible pollinations. Pollen was harvested from full-blown flowers 1 day before the experiment. For castration, buds with anthocyanin were used (the day before the flower opened). To avoid self-pollination, anthers were removed from the flowers and the flowers were placed into gauze isolation bags. The Ac-DEVD-CHO caspase-3 inhibitor was applied and flowers were pollinated on the next day after castration.

#### Treatments with Ac-DEVD-CHO caspase-3 inhibitor

Ac-DEVD-CHO inhibition solution (5 μl; EMD Biosciences and BIOMOL, USA) was applied to the stigmas at concentrations of 0.25, 0.5, 1, and 1.99 mM 2 h before pollination, 2 h after pollination, or during pollination. Unpollinated flowers were the control in this experiment; distilled water (5 μl) was applied to their stigmas. The material (pollinated pistils) was harvested and fixed 2, 4, 6, and 24 h after pollination. For fluorescence microscopy, the pistils were fixed in acetic alcohol (90% ethanol and acetic acid at a ratio of 3 : 1). The pistils intended for assaying the CLP activity and DNA degradation were placed into liquid nitrogen and stored at –70 °C for further isolation of total protein and DNA, respectively. Part of the pollinated flowers was retained as the control for assessing the rate of seed setting.

### Visualization of the pollen tubes in vivo growing in pistil tissues

#### Aniline blue staining

The visualization of growing PTs with aniline blue staining utilizes the ability of this fluorochrome to bind to the callose contained in the pollen grain wall and PTs.

The pollinated pistils fixed in acetic alcohol (90% ethanol and acetic acid, 3 : 1) were used in the experiments. Pistils were macerated in 20% KOH alcohol solution for 20–40 min, washed twice with distilled water, and stained with 0.01% aniline blue solution for 30–40 min. The stained pistils were placed into a drop of glycerin mixed with water (1 : 1) on a glass slide, covered with a cover glass, gently squashed, and examined using a Zeiss Axioplan (Carl Zeiss, Germany) fluorescence microscope with 365 nm excitation filter and 420 nm emission filter. At least 200 PTs were examined in each variant of the experiment.

#### Transmission electron microscopy

Petunia styles (the upper part of pistil 1.5 cm from the surface of stigma) were fixed in 2.5% solution of glutaraldehyde (Merck, Germany) in 0.1 M Sorensen phosphate buffer (pH 7.2) supplemented with 1.5% sucrose. The plant samples were washed from the fixing mixture and postfixed with 1% ОsО_4_ (Sigma, USA), dehydrated in ethanol with rising concentrations (30, 50, 70, 96, and 100%) in propylene oxide (Fluka, Germany), and embedded in the mixture of epon araldite epoxy resins. Ultrathin sections were made with an LKBV (LKB, Sweden) ultramicrotome. Sections were contrasted with uranyl acetate and lead citrate according to the standard procedure [64]. Sections were examined with an H500 (Hitachi, Japan) electron microscopes at an accelerating voltage of 75 kV. The captured images were digitized (scanned with an Epson Perfection 3170) with a resolution of 600 dpi. The images were processed and arranged in Microsoft Photo Editor and Corel DRAW.

### Detection of PCD

#### DNA isolation and agarose gel electrophoresis

PCD markers were identified by assaying DNA degradation using electrophoresis as the analytical method for separation of DNA fragments.

The harvested plant samples were stored in liquid nitrogen. DNA was isolated from pistils according to standard procedure [65] and separated in 3% agarose gel in 1× TRIS borate buffer.

#### CLP activity assay by spectrophotometry

The caspase-3-like protease activity in pistil extracts was assayed according to the cleavage of fluorogenic substrate Ac-DEVD-AMC (Ac-Asp-GLUVAL Asp-AMC and AMC, 7-amino-4-methylcoumarin; EMD Biosciences and BIOMOL, USA). A sample (50 μl) and 400 μM Ac-DEVD-AMC (50 μl) were placed into a well of a 96-well plate (Costar, USA) to measure fluorescence. The release of the fluorophore by cleavage was recorded (excitation at 360 nm and emission at 460 nm) in a BioTek Synergy H1 (USA) microplate reader. The AMC fluorescence was recorded for 3 h with 10-min intervals. The enzyme activity was calculated using the slope of product concentration versus time. To assay the inhibitors, the samples were supplemented with 50 μM of the peptide inhibitor Ac-DEVD-CHO (EMD Biosciences и BIOMOL, USA) and incubated with the substrate, Ac-DEVD-AMC. See Zakharova et al. [21] for comprehensive description.

#### Imaging of CLP Activity in Growing Pollen Tubes via Fluorescence Technique

The CLP activity was assayed using a protocol modified for an in vivo pollen–pistil system. The caspase-3/7-DEVDase activity in living pollen tubes was visualized with the Image-iT™ LIVE Green Caspase-3 and -7 Detection Kit (Invitrogen, Thermo Fisher Scientific, USA). We sampled petunia flowers over 2 h after self-incompatible pollinations (without Ac-DEVD-CHO caspase-3 inhibitor treatment and after Ac-DEVD-CHO caspase-3 inhibitor treatment). Very thin longitudinal sections (4 mm long) of pistil (stigma and the top of style) were made and labeled according to the manufacturer’s protocol. The sections were placed into 1.5-ml Eppendorf tubes; immediately supplemented with the working solution, FAM-DEVD-FMK FLICA reagent, in PBS (pH 7.4) to completely cover the sections; and incubated at ambient temperature in the dark for 60 min. The sections were then gently removed from FAM-DEVD-FMK FLICA reagent, immersed into 5 mM propidium iodide in PBS (pH 7.4), incubated at ambient temperature in the dark for 10 min, rinsed twice with wash buffer, placed on a microscope slide in a drop of wash buffer, covered with a coverslip, slightly crushed, and immediately examined. The pistil sections were imaged with an Axio Scope A1 (Carl Zeiss, Germany) fluorescence microscope with 488/530 nm and 535/617 nm filter sets. The pictures were taken with an Axio Cam Mrm Zeiss camera (Carl Zeiss, Germany) and contrasted using ZEN lite Digital Imaging Software. For each variant of pollination, five individual pistils were stained and imaged. The green fluorescence signal is a direct measure of the amount of an active caspase that was present at the moment when the reagent was added. This signal in the pollen tubes indicated the presence of caspase-3 and caspase-7 activities in the cell. In each variant of the experiment, the number of pollen tubes exceeded 200.

### Statistical data processing

The experiments were performed in three–five biological replicates. Statistical significance was assessed using Student’s *t* test (*P* < 0.05). The data are shown as means and their standard errors.

## Data Availability

All data generated or analysed during this study are included in this published article.
